# Clinical outcomes of combination therapy for hepatocellular carcinoma in a predominantly hispanic South Texas population

**DOI:** 10.3389/fonc.2025.1644056

**Published:** 2025-11-18

**Authors:** John Hoverson, Jonathan Qi, Cameron Mehmken, Darian Chiu, Jack Lowry, Sukeshi Patel Arora, Neil Newman

**Affiliations:** University of Texas Health Science Center at San Antonio, San Antonio, TX, United States

**Keywords:** hepatocellular carcinoma, immunotherapy, combination therapy, locoregional therapy, South Texas

## Abstract

**Background/Aim:**

Currently, studies looking at hepatocellular carcinoma treatments with combination immunotherapy and locoregional therapies are limited in scope. Our study aimed to further clarify the impact of combination therapy using immunotherapy and locoregional therapy on mortality in patients with hepatocellular carcinoma.

**Methods:**

A chart review was conducted on patients with hepatocellular carcinoma who had received either immunotherapy or tyrosine kinase inhibitors. Patients were classified into four treatment arms: 1. Patients treated with locoregional therapy and immunotherapy. 2. Patients treated with locoregional therapy and tyrosine kinase inhibitors. 3. Patients treated with immunotherapy but without locoregional therapy. 4. Combination treatment arm containing Arms 1 and 2. The primary objective was overall survival immunotherapy alone versus combination treatments.

**Results:**

A total of 135 patient charts were analyzed in this study. One hundred nine patients had received immunotherapy, and 102 had received locoregional therapy. Cumulative median survival for all patients from the date of diagnosis was 1.55 years. Median survival for the immunotherapy arm was 0.51 years, and median survival for the combination treatment arm was 2.25 years. Results from the Cox proportional hazards regression model comparing the combination treatment groups against the immunotherapy arm found a hazard ratio of 0.21 (0.12-0.39; p<0.05).

**Conclusion:**

In this study of hepatocellular carcinoma, combination treatment groups receiving locoregional therapy with either tyrosine kinase inhibitors or immunotherapy demonstrated improved survival compared to immunotherapy alone. These results highlight the importance of tailored treatment strategies, particularly in patients with preserved liver function.

## Introduction

Hepatocellular carcinoma (HCC) is the most common type of primary liver cancer, accounting for 80% of all cases (1). In 2020, liver and intrahepatic bile duct cancer was the sixth leading cause of cancer-related deaths in the United States and the third leading cause worldwide (2, 3).

### Treatment advances

While tyrosine kinase inhibitors (TKIs) have shown improvement in longevity, their efficacy is limited as control rates of HCC rarely exceeded 50-60% and adverse effects can be quite limiting (4). As a result, there has been a strong push in recent years to focus on immunotherapy for HCC treatment, particularly in patients with unresectable HCC. While many studies were performed to determine the efficacy of immunotherapy in unresectable HCC, the first landmark study to definitively establish efficacy of immunotherapy agents for these patients was the IMbrave150 trial in 2020. This study established combination treatment with the anti-PDL1 antibody, atezolizumab, and the VEGF neutralizing antibody, bevacizumab, as the preferred first line treatment for unresectable HCC, demonstrating improved overall and progression-free survival as compared to patients treated with sorafenib (5). In 2022, an additional landmark study, the HIMALAYA trial, looked at patients with unresectable HCC who were treated with tremelimumab plus durvalumab and found that they had improved overall survival as compared to those on sorafenib though progression-free survival was not significantly improved (6). Several additional studies have demonstrated the efficacy and safety of immunotherapies in unresectable HCC, including, Checkmate 040, CheckMate 459, KEYNOTE-224, KEYNOTE-240, and CHECKMATE-9DW (5–13).

On the other end of the treatment spectrum, a wide array of locoregional therapy (LRT) options exist for the treatment of HCC. These include microwave ablation, radiofrequency ablation, transarterial chemoembolization, yttrium-90, radiation, and surgical resection. While surgical resection or liver transplant remain as definitive therapeutic options for HCC in the setting of cirrhosis, many patients have disease requiring downstaging with neoadjuvant approaches. Although LRT and immunotherapy alone as treatments for HCC have been studied extensively, their combined role in the treatment of primary liver cancer remains relatively undefined (14). While trials such as EMERALD-1 are studying combination transarterial chemoembolization therapy with durvalumab with or without bevacizumab, data on the overall survival (OS) are still pending (15). Several small-scale studies have evaluated the efficacy of immunotherapy and/or TKI therapy in conjunction with LRT in the treatment of HCC. However, these studies are relatively limited in their scope, and more research, particularly comparing LRT and immunotherapy alone, is needed to determine the clinical benefits that combination therapy provides for HCC patients (14).

### This study

Early clinical studies have suggested that combining LRT with immunotherapy can lead to improved tumor response rates and potentially better OS for patients with HCC (16). In an effort to build on these studies, our study aimed to further clarify the impact of combination therapy on morbidity and mortality in HCC patients. In particular, we sought to obtain survival data in a unique predominantly Hispanic South Texas population.

## Methods

### Patients

This study was conducted at a single institution. Patients diagnosed with HCC and managed at the institution between 2020 and 2023 were identified using electronic medical records. This study was conducted in accordance with the ethical principles outlined in the 2013 Declaration of Helsinki and the 2018 Declaration of Istanbul. Approval was obtained from the Institutional Review Board of the University of Texas Health San Antonio (IRB #20230603EX). The need for written informed consent was waived due to the retrospective nature of the study and use of de-identified patient data. Data were extracted from individual patient charts in Epic^®^ and stored securely in REDCap^®^ for analysis. Eligible patients were ≥18 years of age with a confirmed diagnosis of HCC who had received at least one of the following treatment regimens:

1. Immunotherapy alone (IO)
2. Immunotherapy combined with LRT (IO/LRT)
3. TKIs combined with LRT (LRT/TKI)

Immunotherapy included tremelimumab and durvalumab, atezolizumab and bevacizumab, atezolizumab, nivolumab, durvalumab, or pembrolizumab. LRTs included microwave ablation, radiofrequency ablation, transarterial chemoembolization, surgical resection, radiation therapy, or Yttrium-90. TKIs used included sorafenib and lenvatinib. The original sample size collected for this study was 175. 13 patients were removed due to lack of accurate medical records, and an additional 27 patients were removed due to treatment dates that fell outside of the dates of treatment specified in our protocol.

### End points

The coprimary outcomes for this study included OS defined as the time from HCC diagnosis to death from any cause and hazard ratios for treatment arms estimated using Cox proportional hazards regression to compare OS and progression-free survival (PFS) across treatment groups (IO/LRT, LRT/TKI, and IO alone). Patients who were alive at the last follow-up visit were censored at the time of their last documented visit. Secondary endpoints included the impact of portal vein thrombosis (PVT) on OS, association between liver disease severity using Barcelona Clinic Liver Cancer (BCLC) and Child-Pugh (CP) classifications, and survival and distribution of Liver Function Scores (BCLC and CP) across treatment arms. Safety and side-effect profiles were assessed based on the nature, frequency, and severity of adverse events, according to the NCI Common Terminology Criteria for Adverse Events, version 4.0.

### Statistical analysis

Kaplan-Meier survival analysis was performed to estimate OS and PFS across the treatment arms. The patients were categorized into four treatment arms:

1. LRT/IO arm (immunotherapy plus LRT)
2. LRT/TKI arm (LRT plus TKI therapy, no immunotherapy)
3. IO arm (immunotherapy alone, no LRT)

Treatments arms 2 and 3 were then combined for the purposes of the Kaplan-Meier curve into the fourth treatment arm:

4. Combination treatment arm (LRT/IO and LRT/TKI arms taken together)

After checking proportionality assumptions, a Cox regression model was used to compare the hazard ratios of the treatment arms with IO as the control arm. To further evaluate the effect of LRT on survival, an additional Kaplan-Meier analysis and Cox regression model were performed comparing the combined LRT groups (IO/LRT and LRT/TKI) against the IO group. To rule out confounding variables from liver disease, a multinomial Cox proportional hazards regression model was conducted to assess the effect of treatment group and BCLC staging system stage on survival outcomes. Given the small *n* value in the BCLC D category, this group was combined with BCLC C and assessed as BCLC C/D. Chi-square tests were performed with both CP scores and BCLC classifications to determine distribution of their corresponding classifications across treatment arms. Finally, a Cox proportional hazards regression model was conducted on patients with and without PVT to determine hazard ratios between the two. A Kaplan-Meier curve was constructed to visually assess survival outcomes in patients with PVT compared to patients without PVT. All statistical analyses and generation of figures were conducted using R programming software (version 4.4.2), including the survival package (version 3.7.0) (17). A p-value of <0.05 was considered statistically significant.

## Results

### Demographics

A total of 135 patient charts were analyzed in this study. One hundred seventeen patients were male and eighteen were female. Eighty-seven patients were Hispanic, 41 were White, three were Black, two were Asian, and two were unidentified. The average BMI was 30.4. Sixty-seven patients had a history of diabetes mellitus, and 17 had a history of hypertriglyceridemia. Cirrhosis etiology included 84 patients with HCV, 10 with HBV, 59 with alcohol, 41 with non-alcoholic fatty liver disease, and 26 patients with an unspecified cause of liver cirrhosis. The median CP score was six, of which 69 were CP A, 56 were B, and eight were C. The remaining patients did not have lab data available to calculate a CP Score. Twenty patients had a BCLC staging score of A, 52 were B, 56 were C, and four were D. The remaining patients did not have a BCLC score calculated due to the lack of clinical/laboratory variables available in their charts. One hundred nine patients had received immunotherapy, 69 patients had received TKIs, and 102 had received LRT. A detailed analysis of the patient demographics by category (LRT/IO, IO, and LRT/TKI) is given in **Table 1**.

**Table 1:** Subgroup analyses of patient demographics and medical conditions by treatment group.

Variable	IO	LRT/TKI	LRT/IO
Sex			
Male	27 (81.8)	21 (80.8)	69 (90.8)
Female	6 (18.2)	5 (19.2)	7 (9.2)
Child-Pugh Class			
A	15 (45.5)	10 (38.5)	44 (57.9)
B	16 (48.5)	11 (42.3)	29 (38.2)
C	1 (3.03)	4 (15.4)	3 (3.9)
BCLC			
A	6 (18.2)	4 (15.4)	10 (13.2)
B	10 (30.3)	8 (30.8)	34 (44.7)
C/D	16 (48.5)	12 (46.2)	32 (42.1)
Cause of HCC			
HCV	19 (57.6)	20 (76.9)	49 (64.5)
HBV	2 (6.1)	1 (3.8)	9 (11.8)
MASLD	9 (27.2)	10 (38.5)	24 (31.6)
Alcohol	15 (45.4)	12 (46.2)	36 (47.3)
Other	5 (15.2)	4 (15.4)	16 (21.1)
Extrahepatic Disease	10 (30.3)	13 (50.0)	33 (43.4)
Portal Vein thrombosis	18 (54.5)	7 (26.9)	32 (42.1)
Main portal vein involvement	1 (3.0)	1 (3.8)	13 (17.1)
Treatment			
Ablation		16 (61.5)	27 (35.6)
Chemo-embolization		13 (50.0)	49 (64.5)
Yttrium-90		6 (23.1)	28 (36.8)
Radiation		6 (23.1)	20 (26.3)

†Data are presented as no. (%).

### Survival data

**Figure 1:**
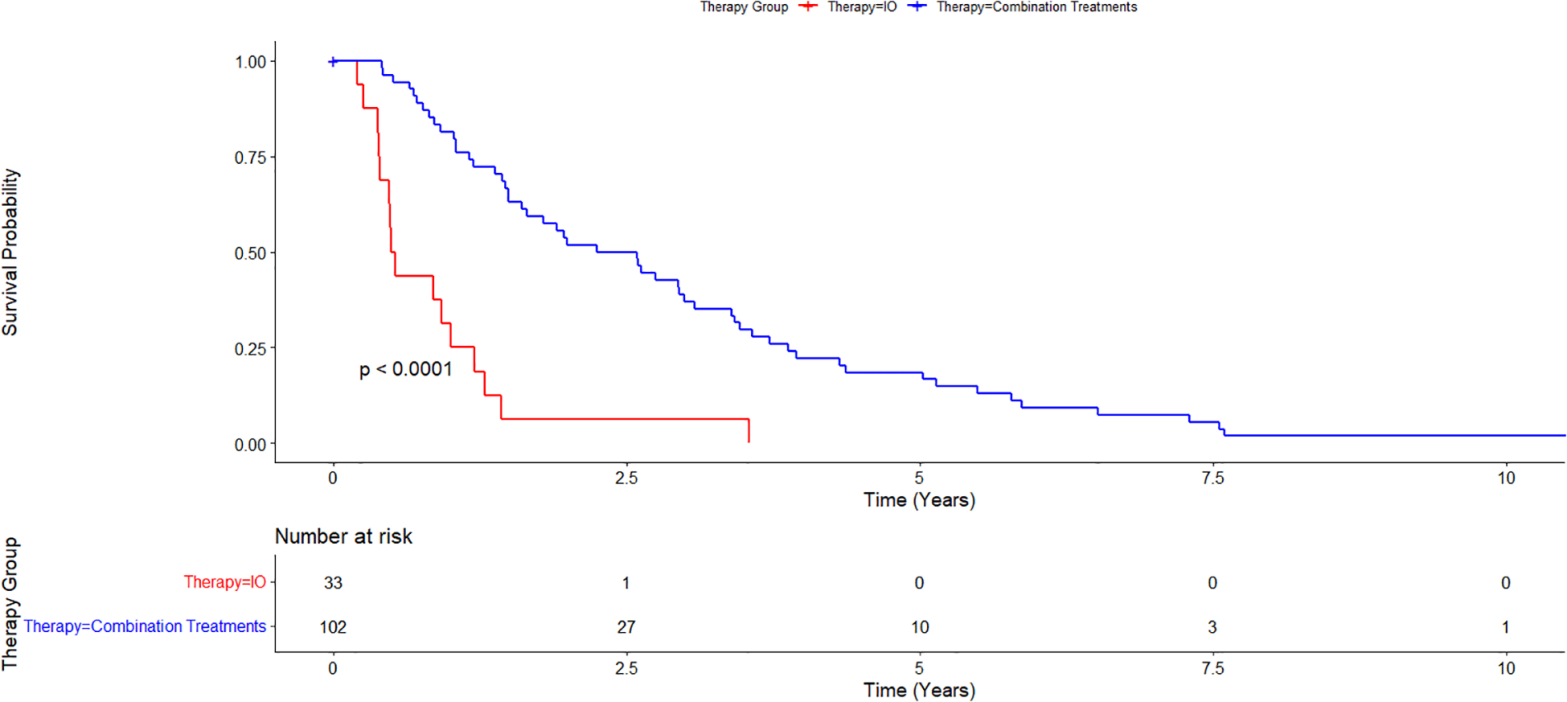
Kaplan-Meier curve comparing immunotherapy alone (IO) versus combination treatment (locoregional therapy + immunotherapy or tyrosine kinase inhibitors). 102 patients were in the combination treatments arm while 33 patients were in the immunotherapy alone treatment arm. Overall survival for the IO treatment arm was 0.51 years and for the combination treatment arm was 2.25 years.

**Figure 2:**
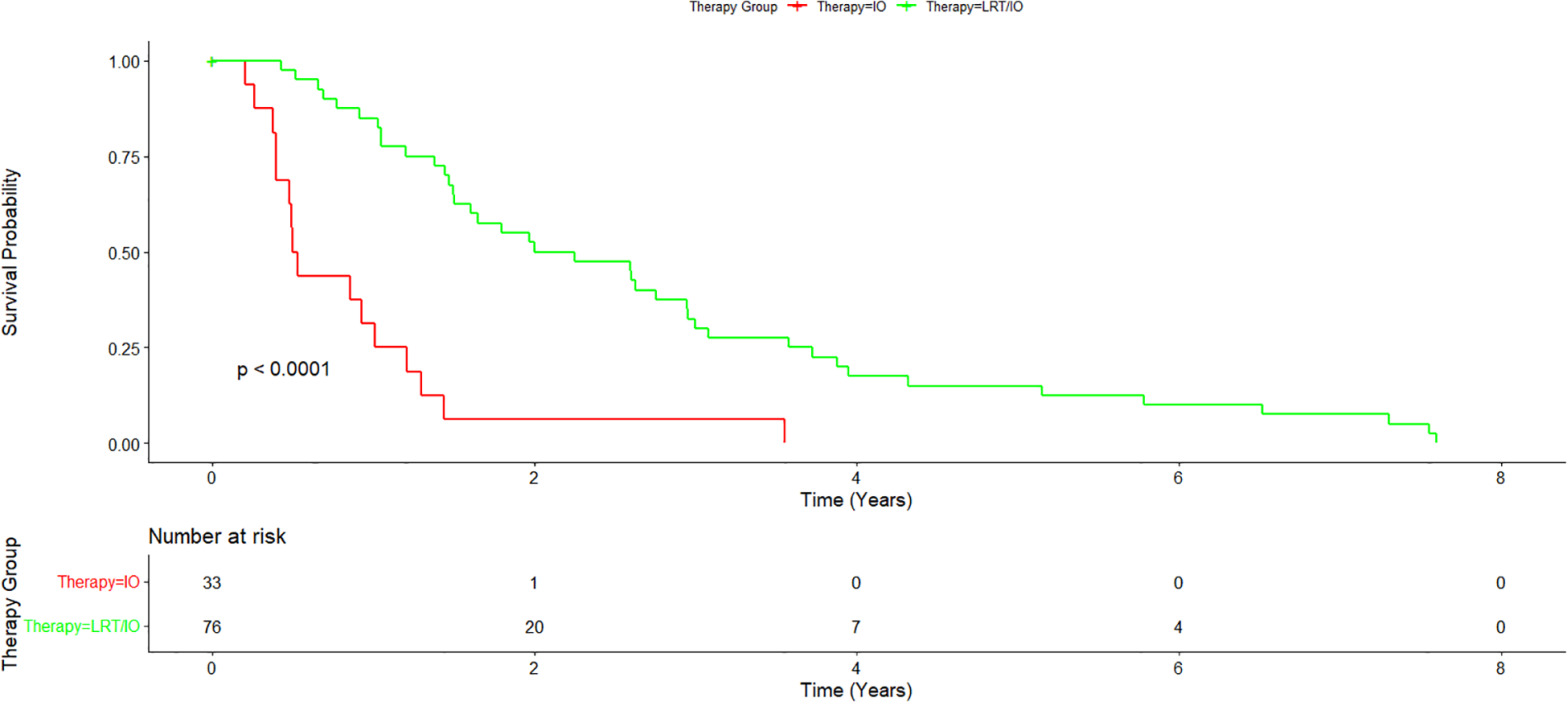
Subset Kaplan-Meier curve comparing immunotherapy alone vs immunotherapy plus locoregional therapy. A subset of the combination treatment arm was further analyzed to compare immunotherapy alone with immunotherapy plus locoregional therapy. Overall survival for the immunotherapy plus locoregional therapy arm was 2.12 years.

Cumulative median survival for all patients from the date of diagnosis was 1.55 years. Median survival from the time of diagnosis for the IO/LRT treatment arm was 2.12 years, median survival for the LRT/TKI treatment arm was 3.41 years, and median survival for the IO treatment arm was 0.51 years. Combined median survival for all groups with IO (LRT/IO and IO) was 1.46 and combined median survival for all groups with LRT (LRT/IO, and LRT/TKI) was 2.25 years. Cox-regression curves comparing the LRT/IO and LRT/TKI arms with the IO arm as a control showed a hazard ratio of 0.23 (0.12-0.43; p<0.05) and 0.17 (0.08-0.38; p<0.05), respectively. Results from the Cox proportional hazards regression model comparing the combination LRT groups (LRT/IO and LRT/TKI) against the IO group found a hazard ratio of 0.21 (0.12-0.39; p<0.05). The Kaplan-Meier curve results for the IO treatment arm versus the combination treatment arms (LRT/IO and LRT/TKI) are shown in **Figure 1**. A subset Kaplan-Meier curve comparing the IO vs LRT/IO treatment arms is shown in **Figure 2**.

### Barcelona clinic liver cancer classifications

**Figure 3:**
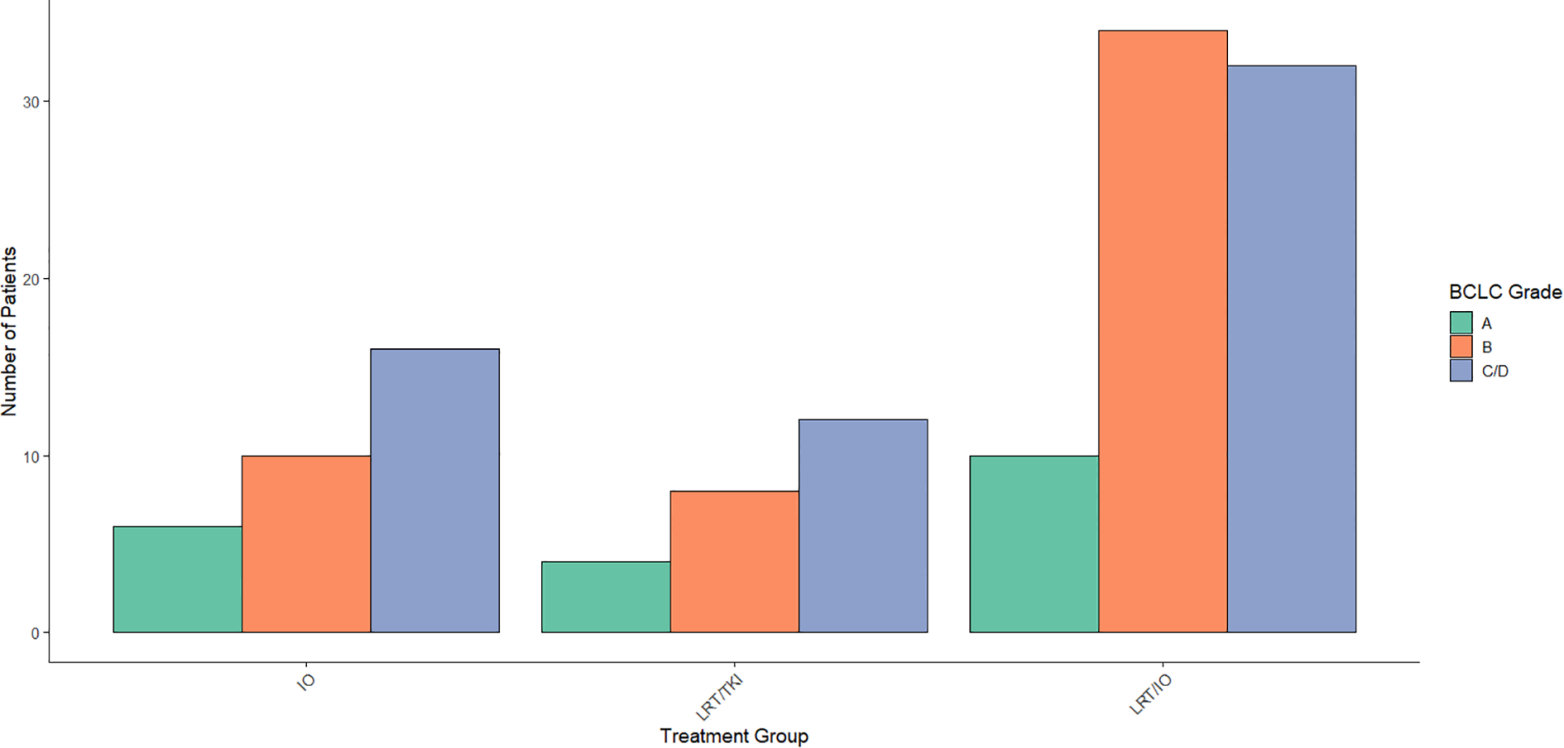
Distribution of Barcelona Clinic Liver Cancer (BCLC) scores across treatment arms. Aggregated data across the treatment arms was compared using a Chi-Square study showed a significant level of BCLC score B patients in the LRT/IO arm, no significant differences in distribution of BCLC scores across treatment arms for scores A, C, or D.

Multinomial logistic regression was performed to evaluate the association between BCLC classification and treatment group allocation, combining BCLC categories C and D into a single group (C/D) due to the small *n* value of BCLC D patients in this study. The reference category for the analysis was BCLC category A. For the comparison between the LRT/TKI treatment group and BCLC category A, the coefficient for BCLC category B was -1.86 (p = 0.04), indicating a significant association between BCLC category B and a reduced likelihood of being allocated to the LRT/TKI treatment group compared to BCLC category A. No significant association was found between the LRT/TKI treatment group and BCLC category C/D (coefficient = -0.97, p = 0.21). For the comparison between the IO/LRT treatment group and BCLC category A, the coefficient for BCLC category B was -0.11 (p = 0.89), suggesting no significant association. Similarly, there was no significant association between BCLC category C/D (coefficient = -0.74, p = 0.31) in the IO/LRT group. These findings suggest that BCLC category B is significantly associated with the LRT/TKI treatment group, but no significant associations were observed for the LRT/IO group. Distribution of BCLC classes by treatment arm are given in **Figure 3**.

To further assess the impact of BCLC stage on survival, a Cox proportional hazards regression was performed with BCLC stages as A, B, and C/D. No statistically significant associations were found between the combined BCLC stages and survival. Specifically, patients with BCLC stage B (HR = 0.818, 95% CI: 0.397–1.686) and combined stages C/D (HR = 1.310, 95% CI: 0.650–2.640) did not show a significant difference in survival compared to stage A (p = 0.585 and p = 0.451, respectively). The concordance index was 0.571, indicating moderate discriminatory ability of the model. The likelihood ratio, Wald test, and log-rank tests all yielded p-values greater than 0.2.

### Child-Pugh analysis

**Figure 4:**
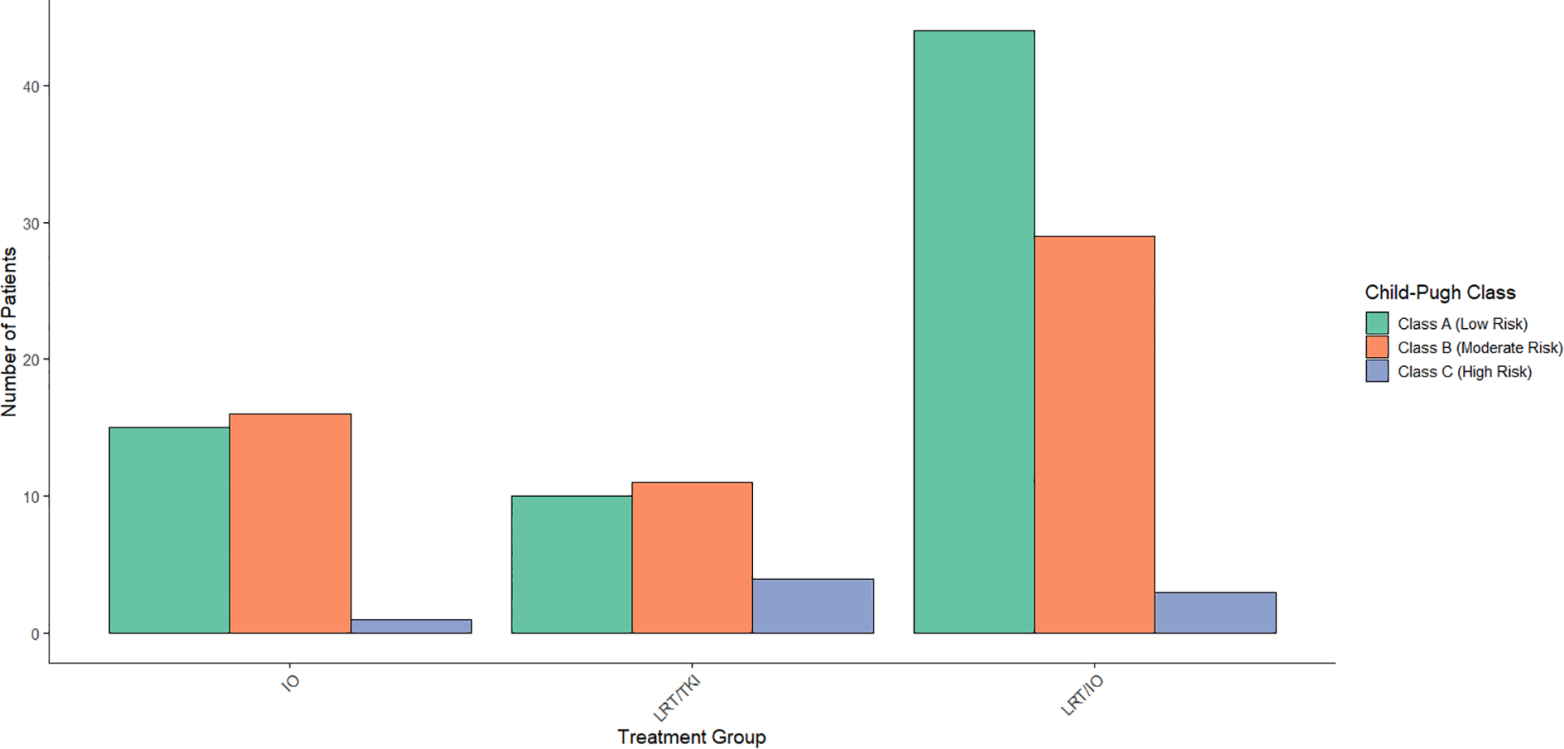
Distribution of Child-Pugh scores across treatment arms. When compared across treatment arms, a Chi-Square study found that none of the Child-Pugh categories were significant predictors of treatment assignment.

A multinomial logistic regression was conducted to assess the relationship between CP classification and treatment group allocation. The results showed that none of the CP categories were significant predictors of treatment assignment. Specifically, no significant associations were found for CP categories 5-10 (p > 0.05 for all). Overall, the CP classification did not significantly affect the treatment group allocation. The distribution of CP classes by treatment arm is shown in **Figure 4**.

A Cox proportional hazards regression analysis was conducted to evaluate the impact of CP risk classification on survival. No significant associations were found between the intermediate (HR = 0.7825, p = 0.399) and high-risk (HR = 0.4412, p = 0.420) groups compared to low-risk patients. The model’s concordance statistic was 0.54, indicating low to moderate discriminatory power, and the proportional hazards assumption was met (p = 0.99). Overall, CP risk classification did not significantly predict survival (p = 0.5 for all tests).

The association between BCLC categories and CP scores was assessed using both a chi-square test and Fisher’s exact test. The chi-square test showed no significant difference in the distribution of BCLC stages across CP scores (*x*²(24) = 23.813, p = 0.4724), indicating that BCLC categories were similarly distributed among the different CP groups. In addition, Fisher’s exact test was performed due to small cell sizes in the contingency table. This test confirmed the findings of the chi-square test, with no significant association observed between BCLC stages and CP scores (p = 0.469).

### Disease progression

**Figure 5:**
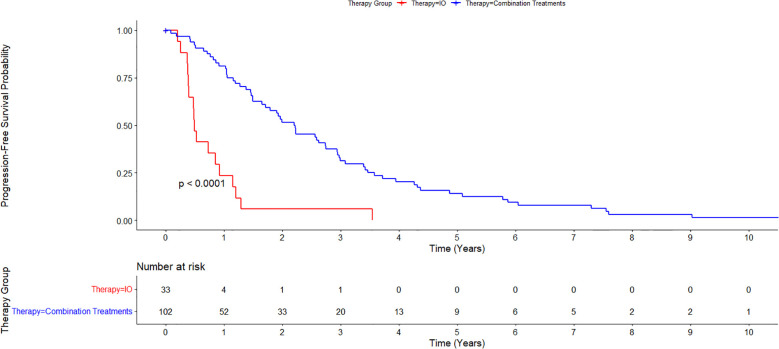
Kaplan-Meier estimate of Progression Free Survival between immunotherapy alone (IO) versus combination treatment (locoregional therapy + immunotherapy or tyrosine kinase inhibitors). Median progression free survival (PFS) for all treatment groups was 1.50 years. Median PFS was 0.487 years for the IO treatment arm, and 2.20 years for the combination treatments group.

The median progression free survival (PFS) in all treatment groups was 1.50 years. Median PFS for each treatment group was 0.487 years for the IO treatment arm, 2.98 years for the LRT/TKI treatment arm, and 2.00 years for the LRT/IO treatment arm. The median PFS in the combination LRT group was 2.20 years. A Cox proportional hazards regression was conducted to compare PFS between the combination LRT group and the IO group. The analysis showed a hazard ratio of 4.54 (95% CI: 2.53–8.15, p < 0.001) for the IO group when compared with the combination treatments. The p-value for this assessment was <0.05. A Kaplan-Meier curve displaying PFS in this patient group is shown in **Figure 5**.

### Portal vein thrombosis

In the OS analysis, a Cox proportional hazards model was used to evaluate the impact of portal vein thrombosis (PVT) on survival. The model revealed a hazard ratio of 1.35 (95% CI: 0.83–2.19), suggesting a modest increased risk of death for patients with PVT compared to those without PVT, although this difference was not statistically significant (p = 0.224). The concordance index was 0.513, indicating a weak ability of the model to discriminate between individuals with and without PVT. The likelihood ratio test (p = 0.2), Wald test (p = 0.2), and log-rank test (p = 0.2) all failed to show significant differences in survival between these two groups.

Analysis using a time-dependent Cox model revealed that the prognostic impact of PVT changed over time. At the time of diagnosis, patients with PVT had a significantly increased risk of death, with a hazard ratio (HR) of 5.99 (95% CI: 1.38–26.04). However, this effect diminished over time, as indicated by the time-dependent interaction term (HR: 0.19, 95% CI: 0.04–0.89). By the 5-year mark, the hazard ratio associated with PVT had decreased to 1.12, suggesting that the prognostic significance of PVT attenuates overt time. The interaction between PVT and time was statistically significant (p = 0.035), confirming a time-varying relationship.

Overall, Kaplan-Meier survival analysis showed no significant difference between patients with and without PVT (p = 0.22), reinforcing the conclusion that the effect of PVT on survival may not be substantial in the long term. However, time-dependent analysis suggests that PVT does have an initial significant impact on survival, particularly in the early years following diagnosis. These findings underline the importance of considering time-varying effects when evaluating the prognostic role of PVT in survival.

### Toxicity data

The observed frequencies of each grade of toxicities in each of the treatment groups are summarized in **Table 2**. A 2x2 Fisher’s exact test was performed for each of four grades of toxicities to determine if the distribution of patients who experienced a toxicity at the specified grade differed significantly between the two treatment groups (IO and combination treatment). The corresponding p-values were then corrected using the Holm-Bonferroni method of false discovery rate correction.

**Table 2:** Incidence of CTCAE toxicities by treatment group.

CTCAE Toxicity Grade	Immuno-therapy alone (IO)	Combination treatments (LRT/IO, LRT/TKI)
Grade 1	23 (59.0)	89 (87.3)
Grade 2	4 (10.3)	27 (26.5)
Grade 3	4 (10.3)	10 (9.8)
Grade 4	0	1 (1.0)

†Data are presented as no. (%).

Overall, patients undergoing combination treatements were more likely to experience low grade toxicities compared to patients undergoing IO. The difference was most pronounced among the number of grade 1 toxicities between the combination group compared to the IO group (87.3% vs 69%, p = .031). However, the differences in grade 1 toxicities failed to reach statistical significance following application of the Holm-Bnferroni method (adjusted p = .124). The combination group also had more grade 2 toxicities compared to the IO group, though this difference was not statistically significant (26.5% vs 12.1%, p = .101). In contrast, patients in the IO group had slightly more grade 3 toxicities compared to those in the combination group (9.8% vs 12.1%, p = .745), and the number of grade 4 toxicities in both groups were infrequent (0% vs 1%, p = 1.000).

## Discussion

### Comparison with current trials

In this study, we examined the efficacy of immunotherapy with and without LRTs as well as LRT in the absence of immunotherapy. A preliminary survey of the data specifically regarding immunotherapy shows comparable median survival rates to the current literature for other groups treated with immunotherapy, particularly in the IMbrave150 and HIMALAYAs trials (5, 6) In our study, the overall median survival for all groups was 1.55 years, which is comparable to those of these trials with median survival rates of 1.1 years in the IMbrave150 trial and 1.38 years in the HIMALAYA trial (5, 6). Compared to prior trials examining combination immunotherapy plus LRT, our data appears to be comparable, or in most cases, improved from prior trials. In comparison to the NCT01853618 trial, which looked at survival rates of patients with tremelimumab in combination with ablation, an OS of 12.3 months was found, which can be compared to our median OS for the IO/LRT treatment arm of 2.12 years (18). Median PFS in the IMBrave150 trial was 6.8 months for atezolizumab–bevacizumab treatment. This can be compared with our data set showing a median PFS of 1.5 years for overall PFS in our study (5). Additionally, PFS in this study at 24 months was comparable to the EMERALD-1 trial which showed a PFS of 15 months in combination IO with LRT (**Figure 1**) (15).

### Overall survival

Several key trends were observed in this study. First, median survival in patients treated with IO and LRT was found to be improved as compared to patients treated with immunotherapy alone, without additional toxicity (per chi-square test results comparing combination treatments versus IO alone). When multinomial logistic regression was used to account for BCLC and CP scores, this trend appeared to remain, indicating that the primary difference in survival outcomes for the various treatment arms was not related to differences in the liver disease progression between the patient groups. The output of the Cox proportional hazards model with an interaction between treatment groups and CP scores was not found to be significant.

This study additionally found that BCLC stage was significantly associated with treatment allocation in the LRT/TKI group, with patients in stage B less likely to receive this treatment compared to those in stage A. No significant associations were observed for the LRT/IO group. While the BCLC stage was not found to be significantly associated with survival outcomes in this study, the analysis was limited by a small patient cohort. The concordance index of 0.571 reflects only modest discriminatory ability. These findings suggest that although BCLC staging may influence treatment selection, its prognostic value for survival may be limited in this setting, underscoring the need for further investigation in larger patient populations.

These findings highlight the importance of considering both the treatment approach and disease severity when evaluating patient outcomes. The inclusion of the BCLC stage in our study helped control for confounding variables and demonstrated that treatment benefits were not merely a reflection of differences in baseline health status among groups. This strengthens the case for the efficacy of treatments in the combination treatment groups and provides a clearer understanding of how disease stage interacts with treatment strategies to influence survival.

### Portal vein thrombosis

The current literature has consistently demonstrated that PVT in the setting of HCC is associated with significantly worse outcomes, particularly in terms of long-term survival, with hazard ratios confirming the increased risk of poor prognosis (19–21) This was supported by our study, which suggested that PVT plays a role in predicting survival, although the hazard ratio decreases over time. Initially, PVT was associated with a significant increase in the hazard of death, with a hazard ratio of 5.99 at baseline, indicating a higher risk for patients with PVT. However, this effect diminished by the 5-year mark, where the hazard ratio dropped to 1.12, but still remained statistically significant. This reduction in the prognostic value of PVT over time is likely due to the increasing mortality burden of HCC. As more patients succumb to HCC over time, the differentiation in survival between those with and without PVT becomes less pronounced. The OS analysis further suggests that PVT is not a significant independent predictor when assessed without considering the time-dependent effect, reinforcing the notion that the impact of PVT on survival may be transient and overshadowed by the high mortality rates associated with advanced liver disease and HCC progression. Additionally, favorable prognostic factors for survival outcomes have been identified in HCC patients with PVT, such as absence of esophageal varices, tumor size, and anatomical resection (22) Patients with these characteristics, along with the impact of treatment modalities, may also contribute to the decreasing hazard ratio over time. Therefore, while PVT may serve as an important early marker, its prognostic value diminishes as patients with HCC approach the end stages of their disease.

### Toxicity data

In our study, we noticed a slightly higher number of grade 2–4 toxicities among patients on combination therapies compared to those on IO. However, none of these differences achieved statistical significance. In particular, the overall incidence and severity of grade 3 or 4 toxicities in this study was consistent with the known profiles of immunotherapy and combination treatments as discussed in other treatment trials (5, 6, 23). The lack of a statistically significant difference in grade 2–4 toxicities suggests that the survival benefits were achieved without a substantial increase in the number of severe adverse events.

The number of grade 1 toxicities in the combination group was increased compared to the IO group by almost 20 percentage points. While this difference was also not statistically significant after false discovery rate correction, the effect size still has clinical ramifications. Grade 1 toxicities, though less severe, can still negatively impact the quality of life of many of these patients. They are also significantly more common and thus have a greater population-level impact. Furthermore, the fact that this study was not fully conducted in the context of a clinical trial raises the possibility that certain lower grade adverse events may have been underreported. While we believe that our survival and toxicity data as well as that of previous studies suggest that the benefits of combination treatment is nontrivial in light of the risks, further research is needed to fully characterize the impact of these treatment approaches on patients’ quality of life and overall treatment experiences.

### Future studies

Future prospective studies should further explore these relationships in larger cohorts and assess the potential mechanisms through which combination therapy via LRT and immunotherapy can benefit patients with HCC. In future studies retrospective studies, we recommend the use of a prospective quality of life assessment using a validated tool specific to hepatocellular carcinoma to fully assess the impact of combination treatments on quality of life.

## Data Availability

The original contributions presented in the study are included in the article/supplementary material. Further inquiries can be directed to the corresponding authors.
